# Self-Consistent Scheme for Spike-Train Power Spectra in Heterogeneous Sparse Networks

**DOI:** 10.3389/fncom.2018.00009

**Published:** 2018-03-02

**Authors:** Rodrigo F. O. Pena, Sebastian Vellmer, Davide Bernardi, Antonio C. Roque, Benjamin Lindner

**Affiliations:** ^1^Laboratório de Sistemas Neurais, Department of Physics, School of Philosophy, Sciences and Letters of Ribeirão Preto, University of São Paulo, São Paulo, Brazil; ^2^Theory of Complex Systems and Neurophysics, Bernstein Center for Computational Neuroscience, Berlin, Germany; ^3^Department of Physics, Humboldt Universität zu Berlin, Berlin, Germany

**Keywords:** complex networks, stochastic models, neural noise, recurrent neural networks, neural dynamics, spike-train statistics, spike-train power spectrum

## Abstract

Recurrent networks of spiking neurons can be in an asynchronous state characterized by low or absent cross-correlations and spike statistics which resemble those of cortical neurons. Although spatial correlations are negligible in this state, neurons can show pronounced temporal correlations in their spike trains that can be quantified by the autocorrelation function or the spike-train power spectrum. Depending on cellular and network parameters, correlations display diverse patterns (ranging from simple refractory-period effects and stochastic oscillations to slow fluctuations) and it is generally not well-understood how these dependencies come about. Previous work has explored how the single-cell correlations in a homogeneous network (excitatory and inhibitory integrate-and-fire neurons with nearly balanced mean recurrent input) can be determined numerically from an iterative single-neuron simulation. Such a scheme is based on the fact that every neuron is driven by the network noise (i.e., the input currents from all its presynaptic partners) but also contributes to the network noise, leading to a self-consistency condition for the input and output spectra. Here we first extend this scheme to homogeneous networks with strong recurrent inhibition and a synaptic filter, in which instabilities of the previous scheme are avoided by an averaging procedure. We then extend the scheme to heterogeneous networks in which (i) different neural subpopulations (e.g., excitatory and inhibitory neurons) have different cellular or connectivity parameters; (ii) the number and strength of the input connections are random (Erdős-Rényi topology) and thus different among neurons. In all heterogeneous cases, neurons are lumped in different classes each of which is represented by a single neuron in the iterative scheme; in addition, we make a Gaussian approximation of the input current to the neuron. These approximations seem to be justified over a broad range of parameters as indicated by comparison with simulation results of large recurrent networks. Our method can help to elucidate how network heterogeneity shapes the asynchronous state in recurrent neural networks.

## 1. Introduction

The autonomous dynamics of recurrent networks of spiking neurons is an important topic in computational neuroscience. Networks of randomly connected excitatory and inhibitory integrate-and-fire (IF) neurons are often used in the study of this problem, because this model is computationally efficient for numerical simulations and even sometimes permits analytical insights (see e.g., Abbott and van Vreeswijk, 1993; Brunel, 2000; Lindner et al., 2005; Richardson, 2009; Deger et al., 2014). Exploring the possible spike statistics in such network models may help us to further our understanding of healthy and pathological neural activity in different brain areas and brain states. Moreover, understanding the autonomous (i.e., spontaneous) activity is also a necessary prerequisite for the comprehension of the network response to external signals and signal transmission and processing capabilities of the network in general.

Recurrent networks of IF neurons can already show a rich repertoire of activity states (Brunel, 2000) shaped by pronounced synchronization and by oscillations on which many computational studies have focused (see e.g., van Vreeswijk et al., 1994; Hopfield and Herz, 1995; Ermentrout et al., 2001; Timme et al., 2006; Ladenbauer et al., 2012). One state that lacks obvious collective effects but still can show a statistically rich behavior is the asynchronous state with low or absent cross-correlations among neurons. This state is found in many network models (van Vreeswijk and Sompolinsky, 1996; Brunel, 2000; Renart et al., 2010; Helias et al., 2014) and also in experimental recordings in different brain areas in the awake and attentive animal (Poulet and Petersen, 2008; Harris and Thiele, 2011).

Although it is frequently assumed in theoretical studies, approximating the asynchronous activity as Poisson spiking with a total lack of temporal correlations is generally not justified. Despite the characteristic absence or weakness of *spatial* correlations among neurons, neural spike trains in the asynchronous state can still show a pronounced *temporal* correlation: experiments have revealed non-flat (i.e., non-Poissonian) spike-train power spectra exhibiting reduced power at low frequency (Edwards et al., 1993; Bair et al., 1994), peaks attained at frequencies close to the firing rate and multiples (Pesaran et al., 2002) or increased power at low frequencies indicating slow fluctuations or bursting (Bair et al., 1994). Some of these features (but also additional ones) have been found for spike-train power spectra from neurons in the sensory periphery (Neiman and Russell, 2011; Grewe et al., 2017) that lack synaptic input from other neurons but are subject to channel noise and other signal-unrelated fluctuations. Theoretically, some (but not all) of these spectral shapes can be already understood if we consider simple stochastic models, e.g., a Poisson process with refractory period (Bair et al., 1994; Jarvis and Mitra, 2001) or, more elaborate, integrate-and-fire models driven by white (Lindner et al., 2002; Richardson, 2008; Vilela and Lindner, 2009b) or colored noise (Middleton et al., 2003; Bauermeister et al., 2013; Droste and Lindner, 2017).

Interestingly, even if completely deterministic neuron models are connected in a random network, corresponding observations of random spiking can be made: the total chaotic input from the network impinging on the single cell acts as an effectively stochastic drive and the resulting spike-train power spectra exhibit in many cases a non-trivial (in particular, non-flat, i.e., non-Poissonian) shape. Depending on cellular parameters as the reset value after spiking (Dummer et al., 2014) or on the strength of synaptic coupling (Ostojic, 2014; Wieland et al., 2015), the spectrum can change drastically (e.g., from strongly peaked spectra to low-frequency dominated spectra with a 1/*f*^α^ form). How spike-train power spectra depend on system parameters in a recurrent network is generally poorly understood [for some effects of presynaptic refractoriness, slow presynaptic rate changes, and short-term synaptic plasticity, see (Schwalger et al., 2015), for effects of the postsynaptic refractory period, see (Bair et al., 1994; Franklin and Bair, 1995)]. Some progress has been achieved though for a related but distinct statistics at a higher modeling level, namely, the power spectrum of the population activity, for which different approximations and numerical schemes have been put forward (Knight, 1972; Brunel and Hakim, 1999; Spiridon and Gerstner, 1999; Mattia and Giudice, 2002; Lindner et al., 2005; Trousdale et al., 2012; Deger et al., 2014; Schwalger et al., 2017). In our paper we focus exclusively on single spike-train power spectra.

According to early work by Mari (2000) and, particularly, by Lerchner et al. (2006), a strong theoretical argument against the white-noise approximation is the self-consistency of the fluctuation statistics. If we think of a homogeneous network of statistically equivalent neurons (identical neural parameters and a fixed number of input connections as in the popular Brunel network; Brunel, 2000), the output statistics of a cell should be related to the input statistics because in the network every driven cell is also a driving cell. In the simple case of current-pulse-coupled IF neurons without a synaptic filter (homogeneous Brunel network), the power spectrum of the input current should be proportional to the power spectrum of the spike train generated by the neuron. As the output power spectrum of a white-noise driven IF neuron is generally not flat (Poisson-like) (Lindner et al., 2002; Vilela and Lindner, 2009a) and, contrary to some claims in the literature, summing many presynaptic spike trains does not remove the temporal correlations of the single process (Lindner, 2006), the flat white-noise spectrum cannot be a self-consistent solution for the network neuron, unless all neurons are poised deep in the subthreshold fluctuation-driven regime of very rare firing (rates are smaller than 1Hz).

The self-consistency of the temporal correlations of input and output in random networks is not an entirely new idea: in statistical physics it has been used to derive correlation functions of disordered spin systems (Sompolinsky and Zippelius, 1982; Eissfeller and Opper, 1992); in neuroscience, it was applied to random networks of coupled rate units by Sompolinsky et al. (1988) (for various recent extensions, see Aljadeff et al., 2015; Kadmon and Sompolinsky, 2015; Mastrogiuseppe and Ostojic, 2017). Generally, the self-consistency condition of the asynchronous state can be employed to determine correlation functions or power spectra without actually simulating the network but by simulating a single element iteratively. If we make a Gaussian approximation for the incoming stream of input spikes, we may ask how correlated (“colored”) this Gaussian noise has to be to evoke a neural spike train with a temporal correlation proportional to that of the driving noise; equivalently, we can ask about the proportionality of power spectra. This idea can be translated into an iterative scheme that finds this solution numerically (if it exists). Such a scheme has first been developed for a spin system (Eissfeller and Opper, 1992); in the neural context it works essentially as follows (Lerchner et al., 2006; Dummer et al., 2014): A single neuron is driven by a Gaussian noise, the output spike train is recorded, its power spectrum is estimated and serves to generate a new Gaussian noise to again stimulate the neuron in the next generation (step in the iterative scheme). Repeating this procedure over a few generations only, for a network with (nearly) balanced recurrent input and moderate synaptic amplitudes, yields an excellent quantitative agreement with the single-cell statistics of a neuron in a large network (Dummer et al., 2014) (Lerchner et al., 2006 used the equivalent correlation function). The simplest version of the procedure fails in the case of strong inhibition and is naturally restricted to homogeneous networks, in which all neurons (excitatory and inhibitory ones) share the same cellular and presynaptic connection parameters. Cortical neural networks are strongly heterogeneous (Meunier et al., 2010; Tomov et al., 2014; Harrison et al., 2015) and, hence, an extension of the method to cases in which neural and connection parameters vary across the network is desirable.

**Figure 1:**
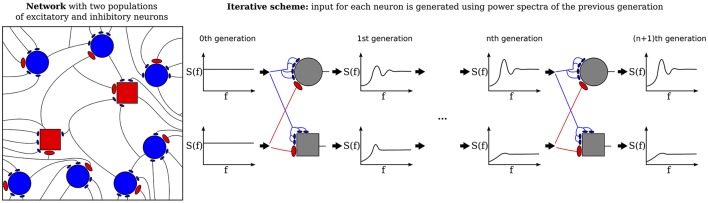
Heterogenous network of excitatory and inhibitory neurons differing in intrinsic parameters. Sketch of the network **(left)** and the corresponding iterative scheme where a single neuron is simulated to represent one population **(right)**. The input of a neuron in the next generation is composed of all power spectra from the previous generations. The power spectrum of each population converges after the nth generation.

The purpose of the present study is to extend the iterative scheme in several directions. First, we develop a simple method to deal with the instability of the iterative scheme at strong recurrent inhibition, which makes the scheme applicable to a much broader range of network parameters. Secondly, as sketched in Figure 1, we study a heterogeneous network, in which excitatory and inhibitory neurons have different parameters (either cellular or with respect to their connectivity) or we consider even several (more than two) populations, which differ in their parameters. As indicated in Figure 1, every population is then represented by a single cell in the iterative scheme, and the input statistics to each cell in a certain generation will be determined from all output spectra of the previous generation.

**Figure 2:**
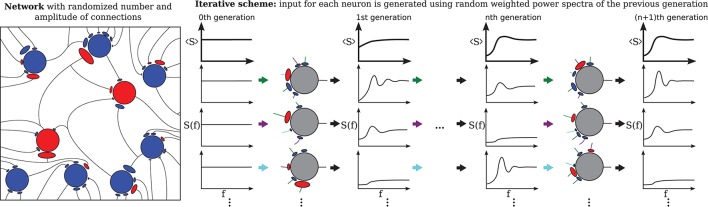
Heterogeneous network with distributed numbers and strengths of synaptic connections (Erdős-Rényi) where the number of presynaptic neurons and the synaptic weights follow a distribution **(left)** and the corresponding iterative scheme where few neurons represent the distributions **(right)**. For each neuron in each generation these parameters are drawn from a distribution and the input is composed randomly from all power spectra from the previous generation. The average power spectrum over all neurons converges after the nth generation.

The third extension, illustrated in Figure 2 concerns the number and strength of synaptic input connections that is in reality certainly not constant and fixed, respectively, as in the Brunel network studied with the iterative scheme by Dummer et al. (2014). Just choosing a simple Erdős-Rényi topology, yields a broad distribution of firing rates and this kind of heterogeneity can be captured in the iterative scheme as well if we simulate a sufficient number of representative neurons, i.e., a sample of the network neurons (cf. Figure 2).

Our motivation for all these extensions of the method is twofold. For once, in cases in which the single-neuron correlation statistics is of interest, e.g., for the emergence of slow fluctuations in recurrent networks (Litwin-Kumar and Doiron, 2012; Ostojic, 2014; Wieland et al., 2015), our extended scheme provides a numerically efficient method that does not require large-network simulations. Hence, if the temporal correlation statistics of the asynchronous state is studied, our results permit to explore the role of network heterogeneity in shaping those correlations. The second purpose of our study is to trigger interest in the self-consistent description of the Gaussian colored noise generated by recurrent spiking networks. Showing that the numerical scheme works in a physiologically relevant parameter regime can also be regarded as a demonstration of the colored-Gaussian-noise approximation's validity and may encourage looking for an analytical description of the network noise via Markovian embedding (Schwalger et al., 2015).

Our paper is organized as follows. Section 2 presents the neuron and network models, introduces important spike-train statistics, shows how to stabilize the iterative procedure such that it works also for strong recurrent inhibition and how to incorporate a synaptic filter, and extends the scheme to the different heterogeneous cases. In section 3, we consider first the fluctuation statistics of the spike trains in the so-called “heterogeneous asynchronous state” of a homogeneous network with strong recurrent inhibition (Ostojic, 2014). Here we demonstrate that slow fluctuations emerge due to their preferred amplification by the network. We review briefly the effect of a synaptic filter and then turn to the different heterogeneous cases. All power spectra found with the iterative scheme are compared to numerical simulations of large and sparse networks. We conclude with a brief discussion of our findings.

## 2. Methods

### 2.1. Neuron model and spike-train statistics

A single neuron is described by the standard leaky integrate-and-fire neuron model (Gerstner et al., 2014). The membrane voltage *v* evolves according to:

(1) $$\tau_{m}\dot{v}\;=\;-v+RI(t).$$

When *v*(*t*) > *v*_*th*_, a spike is emitted and, after a refractory period of τ_*R*_ = 2 ms, the voltage is reset to *v*(*t*) = *v*_*r*_. The parameter τ_*m*_ in Equation (1) is the membrane time constant, which may be different depending on whether the neuron belongs to the excitatory or the inhibitory population. The input current (scaled by the membrane resistance *R*), is denoted by *RI*.

The statistics inspected in this work are based on spike-trains, which are defined as sums of Delta functions

(2) $$x(t)\;=\;\sum_{i}\delta (t-t_{i}),$$

where *t*_*i*_ is the time instant of the *i*th spike. The instantaneous firing rate ν is the (generally time-dependent) average of the spike train, ν = 〈*x*(*t*)〉, and can be determined for a specific neuron within the network by an average over different runs with randomized initial conditions. We are only interested in the asynchronous state, in which ν does not depend on time. In practice, we often average the rate over the population (if appropriate, i.e., if the neurons are statistically equivalent) which is indicated by 〈.〉 (ensemble average) and over time:

(3) $$\nu \;=\;\frac{1}{T}\int_{0}^{T}x(t)dt\;.$$

For the calculation of spectral measures, we define the Fourier transform by

(4) $$\tilde{x}(f)\;=\;\int_{0}^{T}dte^{2\pi ift}x(t),$$

where *T* is our time window and is set in our simulations to *T* = 2 s if not mentioned otherwise. In all simulations we neglect a transient period of 1 s before extracting the statistics over the next *T* = 2 s. The power spectrum of a spike train is then defined by

(5) $$S_{xx}(f)\;=\;\frac{\langle \tilde{x}{\tilde{x}}^{*}\rangle }{T},$$

where ${\tilde{x}}^{*}$ is the complex conjugate of $\tilde{x}$. We note that the power spectrum saturates for infinite frequency at the firing rate, $\underset{f\to \infty }{\lim}S_{xx}(f)=\nu$.

Two important statistical measures can be extracted from the power spectrum. The first is the Fano factor *FF* which is defined as the variance of the spike count $N=\int_{0}^{T}dtx(t)$ over its mean, an expression that can be related to the power spectrum at zero frequency:

(6) $$FF=\frac{\langle \Delta N^{2}\rangle }{\langle N\rangle }=\frac{S_{xx}(f\to 0)}{\nu }.$$

The second statistical measure is the correlation time τ_*c*_. Following Neiman et al. (2007) and Wieland et al. (2015), we consider the spike train's correlation function *c*(τ) = 〈*x*(*t*)*x*(*t* + τ)〉−〈*x*(*t*)〉〈*x*(*t* + τ)〉 (note that here 〈.〉 indicates a time average) and its continuous part ĉ(τ) = *c*(τ)−νδ(τ) to define the correlation time as an integral over the squared and normalized ĉ(τ)

(7) $$\tau_{c}=\int_{-\infty }^{+\infty }d\tau {\left[\frac{\hat{c}(\tau )}{\hat{c}(0)}\right]}^{2}=\int_{-\infty }^{+\infty }df\frac{{\left(S_{xx}(f)-\nu \right)}^{2}}{\nu^{4}},$$

an integral which in turn can be related to an integral over the power spectrum via the Parseval theorem on the right side.

### 2.2. Network model

Different network compositions are studied, many of which are based on the work of Brunel (2000), specifically on his Model B, a heterogeneous random network with fixed in-degree. In contrast to Brunel (2000), we use a larger number of neurons, i.e., an excitatory population size $N_{E}=10^{5}$ instead of $N_{E}=10^{4}$. Independently of the number of populations, there is always a mixture of excitatory to inhibitory neurons with a ratio of 4:1, i.e., *N*_*I*_ = γ*N*_*E*_ where γ = 0.25. Therefore, the total network size is *N* = *N*_*E*_ + *N*_*I*_.

The ℓth neuron from the network has the dynamics

(8) $$\tau_{\alpha }\dot{v_{\ell }}\;=\;-v_{\ell }+R\left(I_{loc,\ell }+I_{ext,\alpha }\right).$$

The external input current to each neuron and its membrane time constant depend on the population it belongs to which is here indicated by index α. The ℓth neuron receives a fixed number of $C_{\ell }^{E}$ ($C_{\ell }^{I}$) excitatory (inhibitory) randomly selected neurons connections from population α = {*E, I*}. The local input is described by:

(9) $$\begin{array}{l} RI_{loc,\ell }(t)=\tau_{\alpha }(\sum_{k=1}^{C_{\ell }^{E}}J_{\ell m_{\ell ,k}}x_{m_{\ell ,k}}(t\;-\;\tau_{D})\\ \;-g_{\alpha }\sum_{i=1}^{C_{\ell }^{I}}J_{\ell n_{\ell ,i}}x_{n_{\ell ,i}}(t\;-\;\tau_{D}))*K(t),\\ \; \end{array}$$

where *g*_α_ is the ratio between average inhibitory and average excitatory synaptic weights, which depends via α on the target neuron (α ∈ *E, I*) The number of presynaptic neurons $C_{\ell }^{E,I}$ will be constant in some cases (fixed in-degree, in sections 2.3 and 2.4 as well as from 3.1 to 3.3) and random in others (as a consequence of a Erdős-Rényi topology in sections 2.5 and 3.4). The excitatory (inhibitory) input neurons are picked randomly from the network and the set of the neuron indexes is denoted by *m*_ℓ,*k*_ and *n*_ℓ,*i*_. The synaptic coupling strength (also called synaptic weight or synaptic efficacy) will be either constant, *J*_ℓ*j*_ = *J*, (in sections 2.3–3.4) or exponentially distributed with mean value 〈*J*_ℓ*j*_〉 = *J* (in section 3.4). We fix the transmission delay at τ_*D*_ = 1.5 ms unless otherwise indicated, and *K*(*t*) is a an optional synaptic filter. In most cases, the filter is not used, which means *K*(*t*) = δ(*t*). Otherwise, it is a simple exponential filter:

(10) $$K(t)=\theta (t)\frac{\exp\left(-t/\tau_{s}\right)}{\tau_{s}},$$

where θ(*t*) is the Heaviside function and τ_*s*_ is the synaptic filter time. Note that in the limit τ_*s*_ → 0, the case without synaptic filter is recovered. If not explicitly stated otherwise, we use the parameter values that are given in Table 1.

**Table 1:** Summary of standard parameters for the iterative scheme with different populations.

**PARAMETERS**		
**Name**	**Value**	**Description**
**NETWORK CONNECTIVITY PARAMETERS**		
*N*_*E*_	10^5^	Size of excitatory population
*N*_*I*_	γ*N*_*E*_	Size of inhibitory population where γ = 0.25
*C*^*E*^	1,000	Number of excitatory synapses per neuron
*C*^*I*^	γ*C*^*E*^	Number of inhibitory synapses per neuron
**NEURON PARAMETERS**		
*v*_*th*_	20 mV	Firing threshold
*v*_*t*_	10 mV	Reset potential
τ_*R*_	2 ms	Refractory period
*RI*_*ext*_	30 mV	External input

### 2.3. Self-consistent scheme for a homogeneous population—stabilization of the scheme for strong recurrent inhibition

The iterative self-consistent scheme developed by Lerchner et al. (2006) and Dummer et al. (2014) is able to reproduce the single-spike-train power spectrum for homogeneous populations close to the balanced regime. In this procedure, in one generation a single neuron is stimulated with a colored noise over many trials, the power spectrum of its spike train is estimated, and using this spectrum and the output firing rate, a new surrogate colored Gaussian noise is generated which is used as the stimulus in the next generation. This procedure is repeated iteratively until the mean value and the spectrum of the driving noise matches in a self-consistent manner approximately the firing rate and the power spectrum of the resulting spike train. We present in detail the heterogeneous iterative self-consistent scheme in section 2.4, for further details of the homogeneous scheme we refer to Dummer et al. (2014).

The version of the scheme by Lerchner et al. (2006) and Dummer et al. (2014) is unable to reproduce self-consistently the statistics of single neurons in a recurrent network with strong relative inhibition *g*. More specifically, in cases where the inhibition is high, the scheme loses stability and the measured firing rate ν oscillates as a function of the generations (Dummer et al., 2014) (a numerical instability in the balanced case is reported in Lerchner et al., 2006, which is unrelated to the instability at strong recurrent inhibition). As a result, network regimes of low firing rate (such as those seen in cortex) cannot be captured.

Here, we propose a method to ensure convergence even with strong recurrent inhibition. We observed that the firing rate oscillations are around the target firing rate, therefore we can use the average firing rate over all past *n* generations as input to the next generation:

(11) $${\hat{\nu }}_{n}=\frac{1}{n}\sum_{q=1}^{n}\nu_{q}.$$

**Figure 3:**
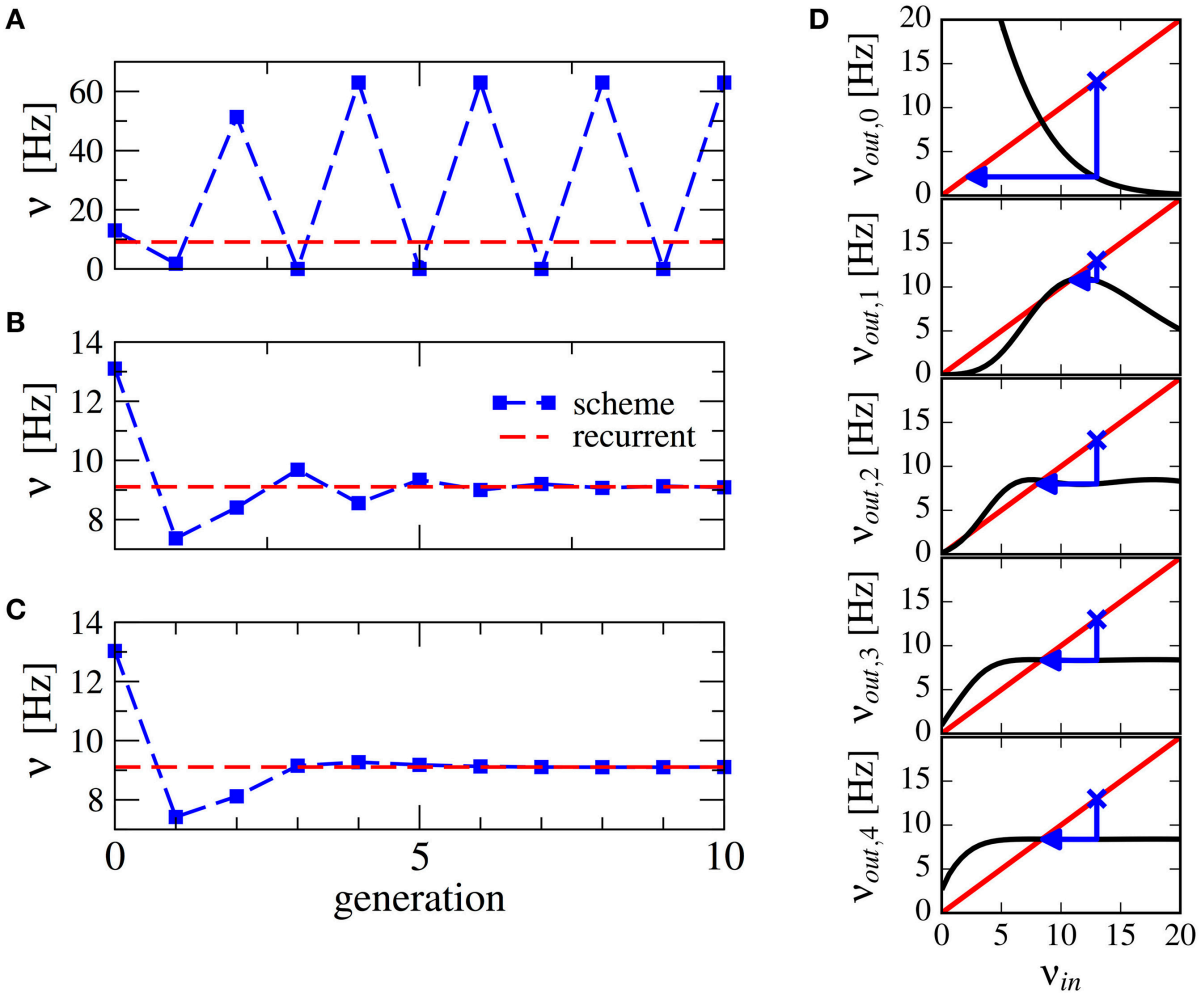
Stabilization of the iterative scheme by averaging over previous generations (inhibition-dominated regime). Convergence of the firing rate in the iterative scheme (blue line) using different procedures. Parameters are *g* = 5.5, *J* = 0.2 mV, τ_*s*_ = 10 ms, γ = 0.25, and *RI*_*ext*_ = 30 mV. Recurrent network (red line) is firing at ν = 9.1 Hz. **(A)** No average is considered, only the previous generation. **(B)** Firing rate is averaged over the past two generations. **(C)** Firing rate is averaged over all past generations. **(D)** Visualization of the averaging procedure: firing rate resulting from *i* iterations of the averaging procedure ν_*out,i*_ of a neuron driven by the firing rate ν_*in*_ (see text). The function ν_*out*_(ν_*in*_) is approximated using the expression for a LIF neuron driven by synaptically filtered white noise (Brunel and Sergi, 1998). The fixed point ν_*in*_ = ν_*out*, 1_ is unstable because |*dν*_*out*_/*dν*_*in*_| > 1 (see Dummer et al., 2014). Few iterations suffice to yield a flat curve indicating a stable fixed point.

This procedure stabilizes the scheme, see Figure 3 for a numerical example. Note that averaging over a higher number of past generations can yield a faster convergence (cf. Figures 3B,C). The effect can be visualized using a similar approach as in Dummer et al. (2014): a map from the input rate to the output rate. We calculate the output rate from the input rate with the approximation for synaptically filtered white noise (Brunel and Sergi, 1998). The effect of the averaging over resulting output rate and input can be captured by the functions ν_*out*,0_ = ν_*out*_(ν_*in*_), $\nu_{out,1}=\nu_{out}\left(\frac{\nu_{in}+\nu_{out}\left(\nu_{in}\right)}{2}\right)$,…, which are shown in Figure 3D. These functions display an increasingly flatter shape in the dependence on the initial firing rate illustrating the stabilizing effect of the averaging.

The procedure of averaging the rate over the past generations will be used only in cases of unstable convergence. Typically, if excitation and inhibition are (nearly) balanced, the scheme is stable and we do not need to apply the averaging procedure.

### 2.4. The self-consistent scheme for several populations

The self-consistent scheme for a homogeneous population can be generalized in different ways. First of all, real networks consist of several types of neurons, that all differ with respect to their physiological parameters. A first important step is to distinguish between excitatory and inhibitory neurons not solely with respect to their postsynaptic effect but to endow inhibitory neurons also with other cellular parameters (membrane time constant, leak potential, mean input current) than excitatory cells. Generally, we distinguish between *P*^*E*^ excitatory and *P*^*I*^ inhibitory populations. In the self-consistent scheme each population is represented by one neuron.

#### 2.4.1. Determination of the second-order statistics

In the situation considered here, every neuron in the network receives a fixed number of inputs. First of all, the mean recurrent input to a given population α is determined by the firing rates of the presynaptic neurons and by the connection parameters in the network:

(12) $$\mu_{\alpha }=\tau_{\alpha }J\left(\sum_{k}^{P^{E}}C_{k}^{E}\nu_{E,k}-\sum_{k}^{P^{I}}g_{k}C_{k}^{I}\nu_{I,k}\right),$$

where ν_*E,k*_ and ν_*I,k*_ are the excitatory and inhibitory firing rates determined by the *k*th presynaptic neuron. Furthermore, by writing the effective input in the Fourier domain, we can obtain the power spectrum of the effective input ${\bar{S}}_{\alpha }\left(f\right)$ to a neuron in the α population given by:

(13) $$\begin{array}{l} {\bar{S}}_{\alpha }(f)=\frac{\langle R\tilde{I}{\left(f\right)}_{\alpha }R{\tilde{I}}^{*}{\left(f\right)}_{\alpha }\rangle }{T}\\ \;=\;\tau_{\alpha }^{2}J^{2}\left(\sum_{k}^{P^{E}}|\tilde{K}(f){|}^{2}C_{k}^{E}S_{k}^{E}(f)+\sum_{l}^{P^{I}}g_{l}^{2}|\tilde{K}(f){|}^{2}C_{l}^{I}S_{l}^{I}(f)\right)\;, \end{array}$$

where $S_{k}^{E}\left(f\right)$ and $S_{l}^{I}\left(f\right)$ are the spike-train power spectra from the *k*th E and *l*th I-cells that provide synaptic input to the population α, respectively, and $\tilde{K}\left(f\right)$ is the Fourier transformed synaptic filter in Equation (10). Note that in order to distinguish it from the output spectra, the input spectra to the population α is identified by a bar, i.e., ${\bar{S}}_{\alpha }$. If more than two populations are present, in Equations (12, 13) their contributions are taken into account by the number *P*^*E*^ and *P*^*I*^ of populations.

#### 2.4.2. Gaussian approximation of the input

We want to use Equations (12, 13) to create an input with the same first- and second-order statistics. For a large number of presynaptic neurons that are only weakly correlated, this statistics will be approximately Gaussian by virtue of the central limit theorem^1^. To generate an input to a neuron embedded in the αth population with a prescribed power spectrum, we generate the Fourier transform

(14) $$R{\tilde{I}}_{G,\alpha }(f)=\sqrt{\frac{{\bar{S}}_{\alpha }(f)}{2\Delta f}}\left({\tilde{\eta }}_{r}+i{\tilde{\eta }}_{i}\right)$$

of a time-dependent function *RI*_*G*,α_(*t*) by drawing two independent Gaussian numbers ${\tilde{\eta }}_{r}$, ${\tilde{\eta }}_{i}$ with unit variance and zero mean in each frequency bin. The frequency resolution is set by the length of the time window, Δ*f* = *T*^−1^. Finally, we generate the time-dependent current *RI*_*G*,α_(*t*) by inverse Fourier transformation of *RĨ*_*G*,α_(*f*).

We start with Gaussian white noise as input as the 0-*th* generation in the scheme and drive *P* neurons, where *P* = *P*^*E*^ + *P*^*I*^ is the number of populations. The neurons are simulated over a number of trials, the output spike-trains are measured and their power spectra, $S_{k}^{E,I}\left(f\right)$, are estimated (1st generation). For the next generation, an input is created using the spike-train power spectra of the first generation in the Gaussian approximation described above. The procedure is repeated until the output power spectra matches the input power spectra, i.e., self-consistency is achieved. In all simulations of the scheme we observed that iterating up to the 30th generation and using 10,000 trials for each generation was enough to reach a self-consistent solution, provided that the scheme converged for the given parameters.

In summary, we simulate the single LIF neuron representing the population α with

(15) $$\tau_{\alpha }{\dot{v}}_{\alpha }=-v_{\alpha }+\mu_{\alpha }+R[I_{ext,\alpha }+I_{G,\alpha }(t)].$$

### 2.5. Self-consistent scheme for a single population with distributed connection parameters

Here, we generalize the iterative scheme to the case where the number of input neurons and the synaptic weights are not the same for the same type of neuron. We deal with a fixed connection probability (Erdős-Rényi topology) and an exponential distribution of synaptic weights, resulting in heterogeneity of firing rates and spike-train spectra. For simplicity, we use the same membrane time constant for inhibitory and excitatory neurons τ_*E*_ = τ_*I*_ = τ = 20 ms and do not use a synaptic filter. We represent the neurons of the network in the iterative scheme by a number of *M* neurons in each generation. The connection parameters of each neuron in each generation are randomly drawn from the assumed distributions. The input to each neuron in the subsequent generation is determined independently from the other neurons taking into account all power spectra of the previous generation.

In case of an Erdős-Rényi topology the numbers of presynaptic neurons are binomially distributed. Likewise, in the scheme we draw binomially distributed random numbers $C_{k}^{E}$ and $C_{k}^{I}$ as numbers of presynaptic neurons connected to neuron α with power spectrum *S*_*k*_(*f*) from the previous generation. The mean of these random numbers is $\langle C_{k}^{E}\rangle =C^{E}/M$ and $\langle C_{k}^{I}\rangle =C^{I}/M$ respectively. In case of exponentially distributed synaptic weights we draw $C_{k}^{E}$ excitatory synaptic weights $J_{k,l}^{E}$ and $C_{k}^{I}$ inhibitory synaptic weights $J_{k,l^{\prime }}^{I}$ for each neuron in the previous generation from the exponential distribution. The input current to the αth neuron is constructed with the mean input

(16) $$\mu_{\alpha }(f)=\tau \sum_{k=1}^{M}\left(\left(\sum_{l\;=\;1}^{C_{k}^{E}}J_{k,l}^{E}-g\sum_{l^{\prime }=1}^{C_{k}^{I}}J_{k,l^{\prime }}^{I}\right)\nu_{k}\right),$$

and with the power spectrum of the effective input that is now taken to be

(17) $${\bar{S}}_{\alpha }(f)=\tau^{2}\sum_{k=1}^{M}\left(\sum_{l\;=\;1}^{C_{k}^{E}}{\left(J_{k,l}^{E}\right)}^{2}+g^{2}\sum_{l^{\prime }=1}^{C_{k}^{I}}{\left(J_{k,l^{\prime }}^{I}\right)}^{2}\right)S_{k}(f).$$

We assume here that *E* and *I* cells have the same spectrum if their connection parameters are equal. With given spectrum and mean input we calculate the voltage dynamics of neuron α as in section 2.4. To adapt the stabilization method described in section 2.3 to the case of a single heterogeneous population described by *M* single neurons, we sum a constant to all firing rates in the *n*-th generation, in order to set its mean firing rate to the average of all generations

(18) $${\hat{\nu }}_{k,n}=\nu_{k,n}-{\langle \nu_{k,n}\rangle }_{k}+\frac{1}{n}\sum_{q=1}^{n}{\langle \nu_{k,q}\rangle }_{k},$$

where ν_*k,n*_ is the firing rate of the *k*-th neuron in generation *n* and ${\hat{\nu }}_{k,n}$ is the transformed firing rate that should be used to calculate the input of the next generation. Note that the mean of the first two terms is zero. These ${\hat{\nu }}_{k,n}$ are used in place of ν_*k*_ in Equation (16) to stabilize the scheme in case of strong inhibition.

For the simulation of these networks, we use the Brian spiking network simulator (Goodman and Brette, 2009).

## 3. Results

### 3.1. Homogeneous network with strong recurrent inhibition and additional synaptic filtering

**Figure 4:**
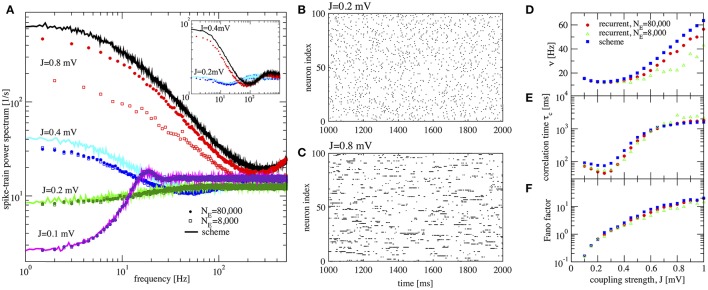
Large amplification of slow fluctuations explains heterogeneous asynchronous state in a homogeneous network. Same set-up as in Ostojic (2014), τ_*E*_ = τ_*I*_ = 20 ms, *RI*_*ext*_ = 24 mV, γ = 0.25, τ_*D*_ = 0.55 ms, *g* = 5, and τ_*R*_ = 0.5 ms. This is a parameter region where the network fires at low-frequency regime and high inhibition *g* = 5 is set. **(A)** Single-neuron spike-train power spectra from recurrent network (circles for *N*_*E*_ = 80,000 and squares for *N*_*E*_ = 8,000) and self-consistent scheme (solid lines) for different values of *J*. Inset: power spectra for a large network (*N*_*E*_ = 100, 000) of exponential IF neurons (single-neuron dynamics is $\tau_{m}\dot{v}=-v+\Delta_{T}\exp\left(\frac{v-v_{th}}{\Delta_{T}}\right)+RI\left(t\right)$ where τ_*E*_ = τ_*I*_ = 20 ms, *RI*_*ext*_ = 30 mV, γ = 0.25, τ_*D*_ = 0.55 ms, *g* = 5, τ_*R*_ = 0.5 ms, and Δ_*T*_ = 0.2) for two different values of the synaptic coupling, showing the same qualitative difference in low-frequency power as the LIF networks. **(B,C)** Raster plots containing 100 neurons from the LIF network with *N*_*E*_ = 80,000 for *J* = 0.2 mV, and *J* = 0.8 mV, respectively. **(D–F)** Firing rate ν, correlation time τ_*c*_, and Fano factor for different values of *J* for both recurrent and self-consistent scheme evaluated in a simulation of *T* = 100 s.

We would like to start with results for the inhibition-dominated network (*g* > 4), in which firing rates are low. In this regime, the iterative scheme as proposed by Dummer et al. (2014) is highly unstable and we only obtain convergence with the averaging procedure described in section 2.3. To demonstrate that the averaging procedure works in such a situation, we consider in Figure 4 the network studied by Ostojic (2014) who found two contrasting asynchronous states when varying the synaptic strength *J*.

In Figure 4A spike-train spectra for strong recurrent inhibition (*g* = 5) for different values of *J* and different network sizes are shown. The power spectra of these network simulations are close to those of the iterative scheme in most cases. For weak coupling, the agreement between spectra is always good; discrepancies for large *J* become smaller with increasing network size because cross-correlations become less important in this limit. An additional reason for discrepancy is that the Gaussian approximation becomes less accurate for strong synaptic strength. The change in spike-train power spectra upon increase of the synaptic coupling does not hinge on the specific nature of the subthreshold function in the IF model. If we replace the leaky IF model by an exponential IF model (Fourcaud-Trocmé et al., 2003) in the network and in the recurrent scheme, we observe a similarly drastic change in low-frequency power if the synaptic strength is doubled (inset of Figure 4A). Also for this single-neuron model the agreement between spectra from the network and from the self-consistent scheme is fairly good.

When the coupling strength *J* increases, the firing rate first decreases and then increases (Figure 4D). More interestingly, with increasing coupling we see a transition from Poisson-like irregular firing (Figure 4B) to bursty firing of single neurons (Figure 4C), i.e., periods of strong firing are separated by pauses. In the latter state one can observe a broad distribution of spike counts, and that is why this state has been referred to as heterogeneous asynchronous state (Ostojic, 2014). In terms of the power spectrum this transition becomes manifest as an amplification at low frequencies (Wieland et al., 2015); correspondingly the Fano factor increases (Figure 4F). Together with the minimum in the correlation time (Figure 4E) (attained at a coupling where the Fano factor is about unity), our results confirm that the transition described by Ostojic (2014) in the inhibition-dominated regime is essentially the same as the one observed and explained by Wieland et al. (2015) for the balanced case *g* = 4.

In summary, the results in Figure 4 indicate that the emergence of a new heterogeneous asynchronous state for strong synaptic coupling can be explained only using the properties of a single neuron and the self-consistency condition, here demonstrated by our iterative single-neuron scheme.

**Figure 5:**
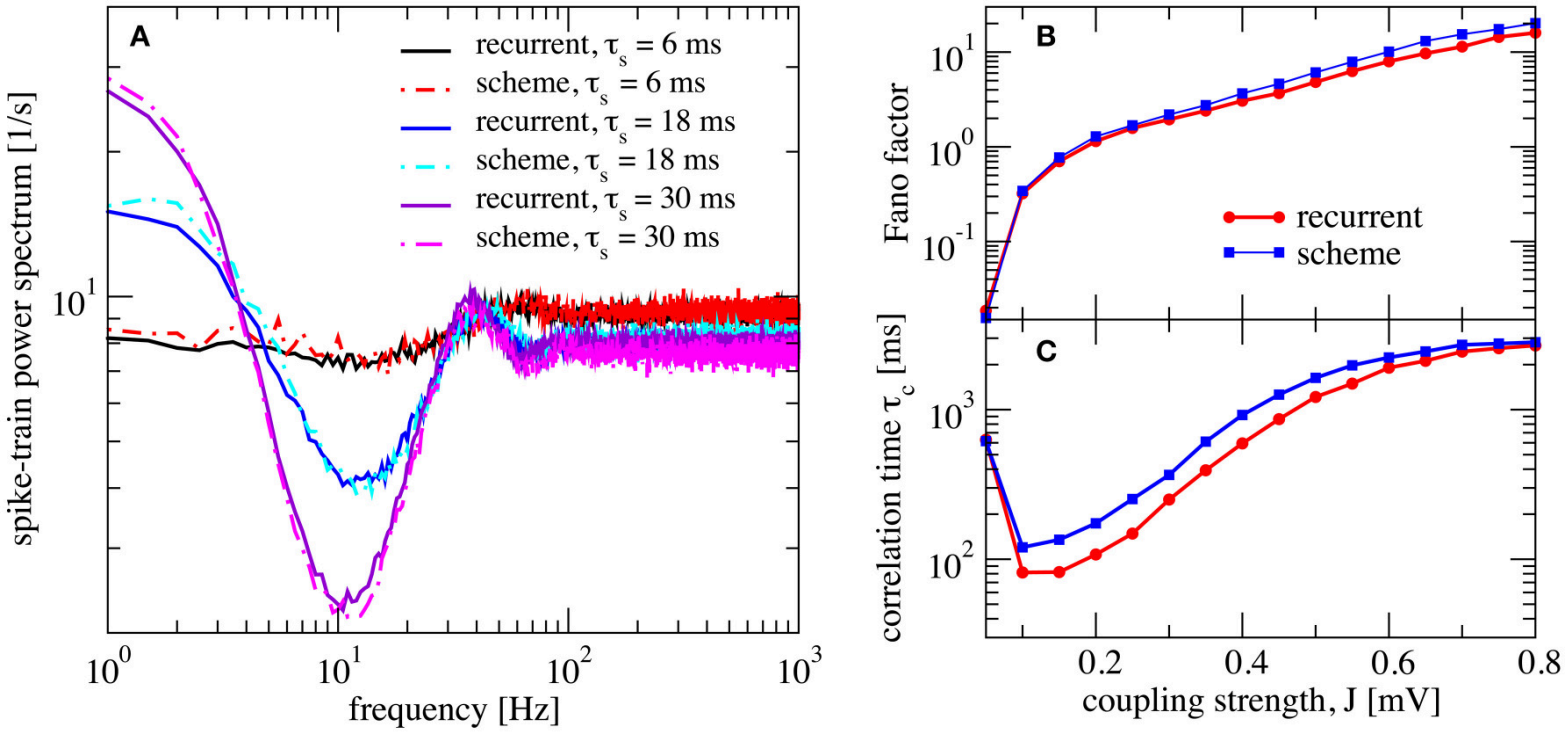
Self-consistent scheme also works for a network with synaptic filtering. **(A)** Effect of τ_*s*_ on the recurrent network and iterative scheme, the plot displays power spectra for different values of τ_*s*_. This is an inhibition-dominated regime with exponential synapses and *J* = 0.2 mV. In this region, the network fires at low firing rates. In **(B,C)** we fix τ_*s*_ at 10 ms and show Fano factor and correlation time τ_*c*_ for different values of *J* for both recurrent and self-consistent scheme. Other parameters as in Figure 4.

We now investigate the effect of a finite synaptic filter, Equation (10). Not surprisingly, a pronounced synaptic filter (large τ_*s*_) leads to a long time scale in the network dynamics, as revealed by the increased power at low frequencies (Figure 5). The synaptic filter, Equation (10) is scaled such that the total charge per input spike remains constant. Therefore, an increased time constant for the exponential decay renders the postsynaptic response smaller in amplitude and longer in duration. This longer duration of postsynaptic responses extends the range of temporal correlations in the input to the neuron, which in turn causes the slow fluctuations in the neuron's activity. The resulting power spectrum (Figure 5A), especially for long synaptic time constant, looks similar to that of a colored-noise driven perfect IF model (see Figure 9 in Middleton et al., 2003). We emphasize that the emergence of the slow time scale is here imposed by the long-lasting synaptic filter which is in marked contrast to the network amplification of slow fluctuations for strong synaptic coupling discussed before in Figure 4.

We also verified that the synaptic filter does not change qualitatively the emergence of slow fluctuations for strong coupling (i.e., the heterogeneous asynchronous state discussed above). Using τ_*s*_ = 10 ms we still see the characteristic strong increase in Fano factor (Figure 5B) and a minimum in the correlation time (Figure 5C).

### 3.2. Networks with different parameters for excitatory and inhibitory neurons

In the following, we return to the limit of instantaneous synapses τ_*s*_ → 0, i.e., *K*(*t*) = δ(*t*), and introduce different parameter values for excitatory neurons (E-cells) and inhibitory neurons (I-cells). First of all, in order to test whether the applicability of the scheme hinges on the exact value of crucial parameters, we choose a small change of the membrane time constant between E and I-cells: τ_*I*_ = 19 ms and τ_*E*_ = 20 ms. Secondly, we make the relative strength of recurrent inhibition, *g*_*E*_ and *g*_*I*_, different for the two populations in order to see whether the generalized iterative scheme with two neurons can cope with this heterogeneous situation.

**Figure 6:**
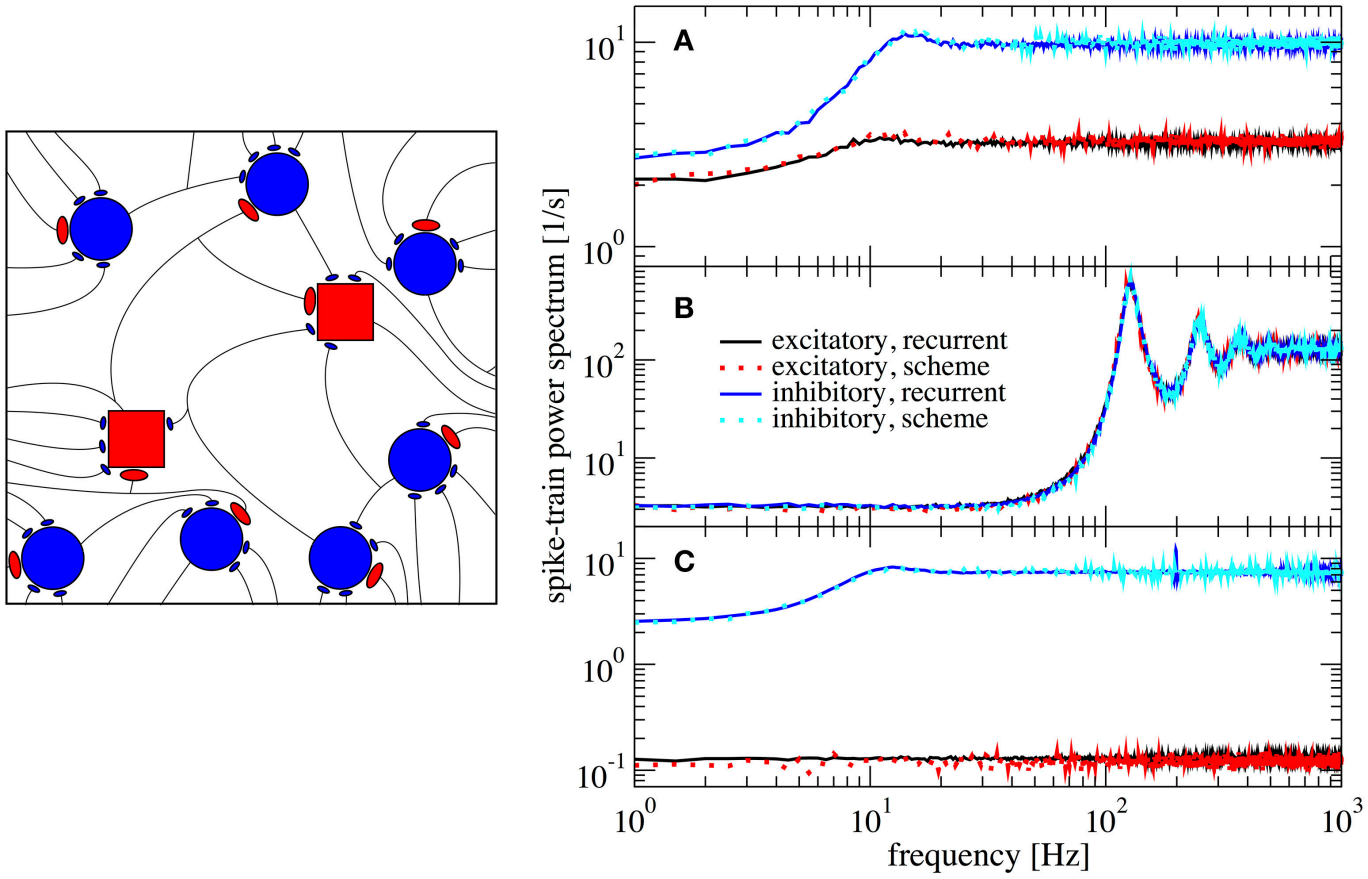
Example spectra for heterogeneous network of excitatory and inhibitory neurons differing in their parameters. Comparison of power spectra of the recurrent network with two different populations and the self-consistent iterative scheme with two neurons. Parameters are in **(A)** (*g*_*E*_, *g*_*I*_) = (4.2, 4.0), in **(B)** (*g*_*E*_, *g*_*I*_) = (3.7, 3.7), and in **(C)** (*g*_*E*_, *g*_*I*_) = (4.25, 3.6).

In Figure 6 we show power spectra obtained from simulations of the recurrent network and of the iterative scheme for different combinations (*g*_*E*_,*g*_*I*_). In Figure 6A the two populations are statistically rather different with an E-cell firing rate of ν_*E*_ = 3.2 Hz whereas I-cells fire at ν_*I*_ = 9.7 Hz. Both spectra are well reproduced by the iterative scheme and show a “green” shape (in the colored noise lingo, this is white minus red noise). That means, the spectra exhibit a dip at low frequencies, but this is much more pronounced for the I-cells. Even when we increase the difference in recurrent inhibition and the two types of neurons fire at lower frequencies of ν_*E*_ = 0.1 Hz and ν_*I*_ = 7.4 Hz, the agreement of the spectra from the iterative scheme and from the network simulations is excellent (Figure 6C). If we choose the relative recurrent inhibition to be the same, the neural dynamics differ only by the small difference of the membrane time constants, which does neither cause significant differences in the firing rates (ν_*E*_ = 128.9 Hz and ν_*I*_ = 129.9 Hz) nor in the shape of the power spectra (cf. Figure 6B).

In order to explore the quality of the approximation systematically, we evaluated the discrepancy using the relative integrated error

(19) $$\Delta =\frac{\int_{0}^{f_{cut}}df{\left(S_{xx,net}(f)-S_{xx,scheme}(f)\right)}^{2}}{\int_{0}^{f_{cut}}dfS_{xx,net}^{2}(f)},$$

where *f*_*cut*_ = 2ν_*I*_ (we use the inhibitory firing rate because it is usually higher).

**Figure 7:**
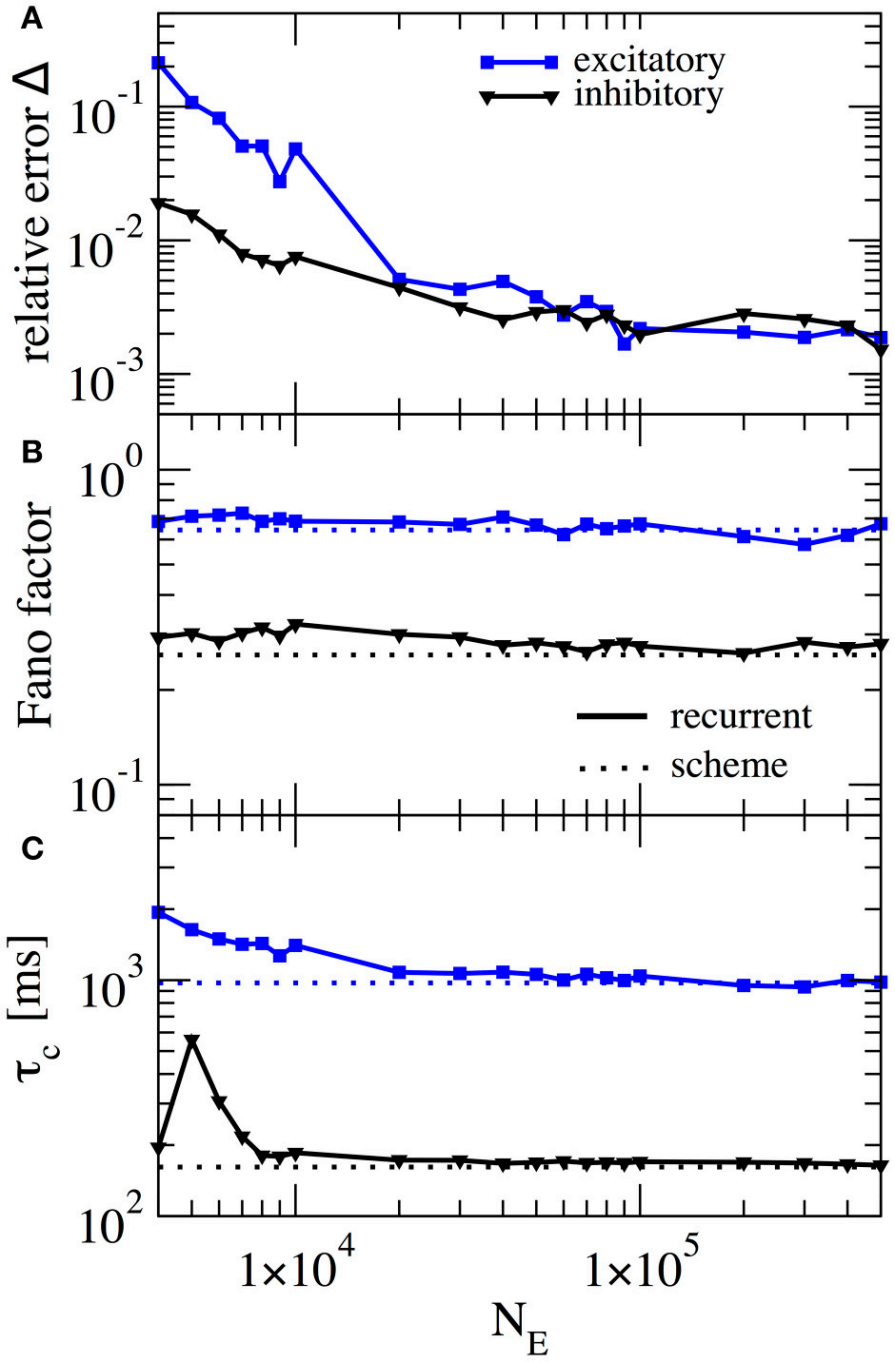
Performance of iterative scheme improves with network size. The performance of the iterative scheme depends on the network size. Curves were produced for the case *g*_*E*_ = 4.2 and *g*_*I*_ = 4.0, same parameters as in Figure 6A. **(A)** Relative error dependence of the scheme for a network size *N*_*E*_ evaluated with the integrated relative error defined in Equation (19). **(B)** Fano factor dependence where dotted line represents the iterative scheme prediction and solid lines the recurrent network. **(C)** correlation time τ_*c*_ dependence.

In our scheme the assumption of weak cross-correlations among neurons in the network is crucial - indeed we assume an infinitely sparse system that is in a perfectly asynchronous state. This is, of course, a somewhat artificial limit and thus it is interesting how, for a fixed number of connections (about 10^3^), the squared deviation as well as important statistics such as the Fano factor depend on the system size. In Figure 7 this dependence is illustrated for the case where *g*_*E*_ = 4.2 and *g*_*I*_ = 4.0, the same parameters as in Figure 6A. For the chosen connectivity, a minimal number of *N*_*E*_ = 20, 000 E-cells seems to be required to reach a good approximation (relative error below 1% for both *E* and *I* cells) with the self-consistent scheme. This plot illustrates that although sparsity is an important assumption for the self-consistent determination of spike-train power spectra, it does not lead to the necessity to consider exorbitantly large networks.

### 3.3. Networks with three distinct populations and distinct modules

In principle, the proposed iterative scheme is applicable to any number of populations. As long as the resulting activity is sufficiently asynchronous (implying weak cross-correlations) and the synaptic strength is not excessively large (needed for the Gaussian approximation), the iterative scheme should converge to a self-consistent result. Here we demonstrate that the extended scheme also works for networks with more than two populations and study two cases: a network with three distinct populations and a modular network.

**Figure 8:**
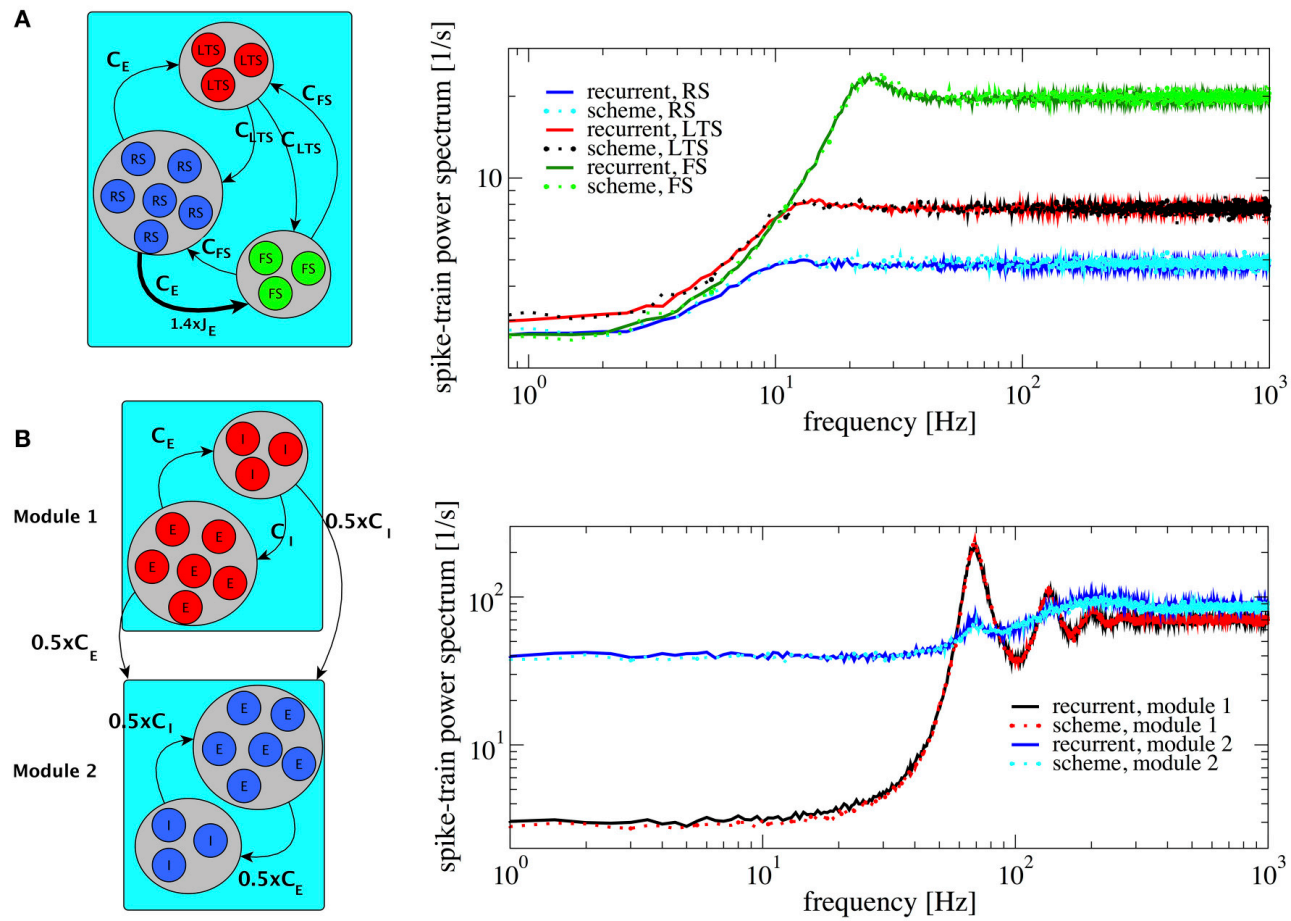
Heterogeneous networks with more than two populations. Comparison between power spectra of the recurrent network (solid lines) with the iterative scheme (dashed lines). **(A)** Three different populations (*RS*, *LTS*, and *FS*) whereby the inhibitory population is composed of *LTS* (*N*_*LTS*_ = 0.25 × *N*_*E*_, *C*_*LTS*_ = 0.25 × *C*_*E*_) and of *FS* (*N*_*FS*_ = 0.05 × *N*_*E*_, *C*_*FS*_ = 0.05 × *C*_*E*_). Parameters are *g* = 4.0, τ_*RS*_ = 20 ms, τ_*LTS*_ = 19 ms, τ_*FS*_ = 21 ms. We multiply the synaptic strength by 1.4 for the excitatory weight to inhibitory neuron from population 2. **(B)** Modular network, modules 1 and 2 communicate through connections represented at the sketch in the left. Each module contains 1.25 × 10^5^ cells with E:I ratio 4:1. In both modules τ = 20 ms. At module 1 *J* = 0.1 mV and at module 2 *J* = 0.27 mV, for all modules *g* = 4.0. Module 2 exhibits a spectral hump around 80 Hz which comes from the interaction with module 1.

An example of three populations is given by a combination of one excitatory and two inhibitory populations (Figure 8A), biologically inspired by a cortical network with excitatory regular spiking neurons (RS), inhibitory fast-spiking (FS), and low-threshold spiking (LTS) neurons (see Izhikevich, 2003; Tomov et al., 2014 and references therein). Their firing rates are ordered such that ν_*FS*_ > ν_*LTS*_ > ν_*RS*_. This heterogeneous situation is achieved by changing both membrane time constants, which are chosen to be τ_*FS*_ = 21 ms, τ_*LTS*_ = 20 ms, and τ_*RS*_ = 19 ms, and making one of the synaptic weights in the network (connecting RS neurons to FS neurons) 1.4 times stronger (indicated by the thick arrow in Figure 8A, left). This setting illustrates how heterogeneity of connectivity and membrane time constants shape the power spectra statistics.

The resulting spectra are well-captured by the iterative scheme; they all display the effect of neural refractoriness by the dip at low frequencies (Bair et al., 1994; Franklin and Bair, 1995) but to a different degree. The dip is most pronounced for the fast spiking neurons; the regular spiking neurons fire with a statistics that is closest to a Poisson process with a flat power spectrum.

According to a common view, the cortex possesses a modular structure (Boucsein et al., 2011; Tomov et al., 2014, 2016), a feature that we take into account in the next setup. We consider two different modules as shown in Figure 8B. The two modules are equal to each other with respect to the population size and each consists of an E-I network with *N*_*E*_ = 100, 000 and membrane time constants τ = 20 ms, requiring the simulation of two neurons in total in the self-consistent scheme. For module 1 and 2 we choose *J* = 0.1 mV and *J* = 0.27 mV, respectively. With this choice, the two modules operate in different regimes: module 1 in a fast-fluctuation mode with low Fano factor and peaked power spectrum, module 2 in a regime of dominating slow fluctuations (cf. section 3.1). In module 2, we rewired 50% of the connections so that they come from module 1, i.e., it receives 0.5*C*_*E*_ excitatory inputs and 0.5*C*_*I*_ inhibitory inputs from module 1. This results in a highly heterogeneous situation which is reflected in the power spectrum of module 2: in contrast to the behavior observed in Figure 4, the power spectrum of module 2 contains an additional hump around 80 Hz. The power spectra of all different neurons in this setup are well represented by the iterative scheme, cf. Figure 8B.

The result in Figure 8B demonstrates that the iterative scheme can capture complex situations involving the interaction among different modules. The simulated network contained in total 250,000 neurons and the iterative scheme reproduced the single neuron correlation statistics with high accuracy using only two neurons.

### 3.4. Network with randomized number and weight of synaptic inputs

Here we use the iterative scheme to represent network dynamics for distributed connection parameters in a single population. As a first example, we consider a network in which connections are made with a fixed probability (Erdos and Rényi, 1960) instead of a fixed in-degree (as in all previous examples). This Erdős-Rényi topology yields a binomially distributed in-degree which is certainly a more realistic scenario than the fixed in-degree. Furthermore, a distributed in-degree leads to a distribution of firing rates, as seen in cortical networks (Griffith and Horn, 1966; Koch and Fuster, 1989; Shafi et al., 2007; Hromádka et al., 2008; O'Connor et al., 2010; Roxin et al., 2011). In addition to a random number of connections, in cortical networks also the synaptic weights are not fixed but follow a long-tailed distribution (Song et al., 2005; Gilson and Fukai, 2011). This feature can be approximated in the recurrent network model by drawing the weights from an exponential distribution.

In this version of the iterative scheme, we have *M* neurons that represent independent samples of the distributions of excitatory and inhibitory in-degrees and weights. Consequently, the firing rates and power spectra of the *M* neurons will differ and reflect the heterogeneity of these measures in the recurrent network (for further details, see section 2.5).

**Figure 9:**
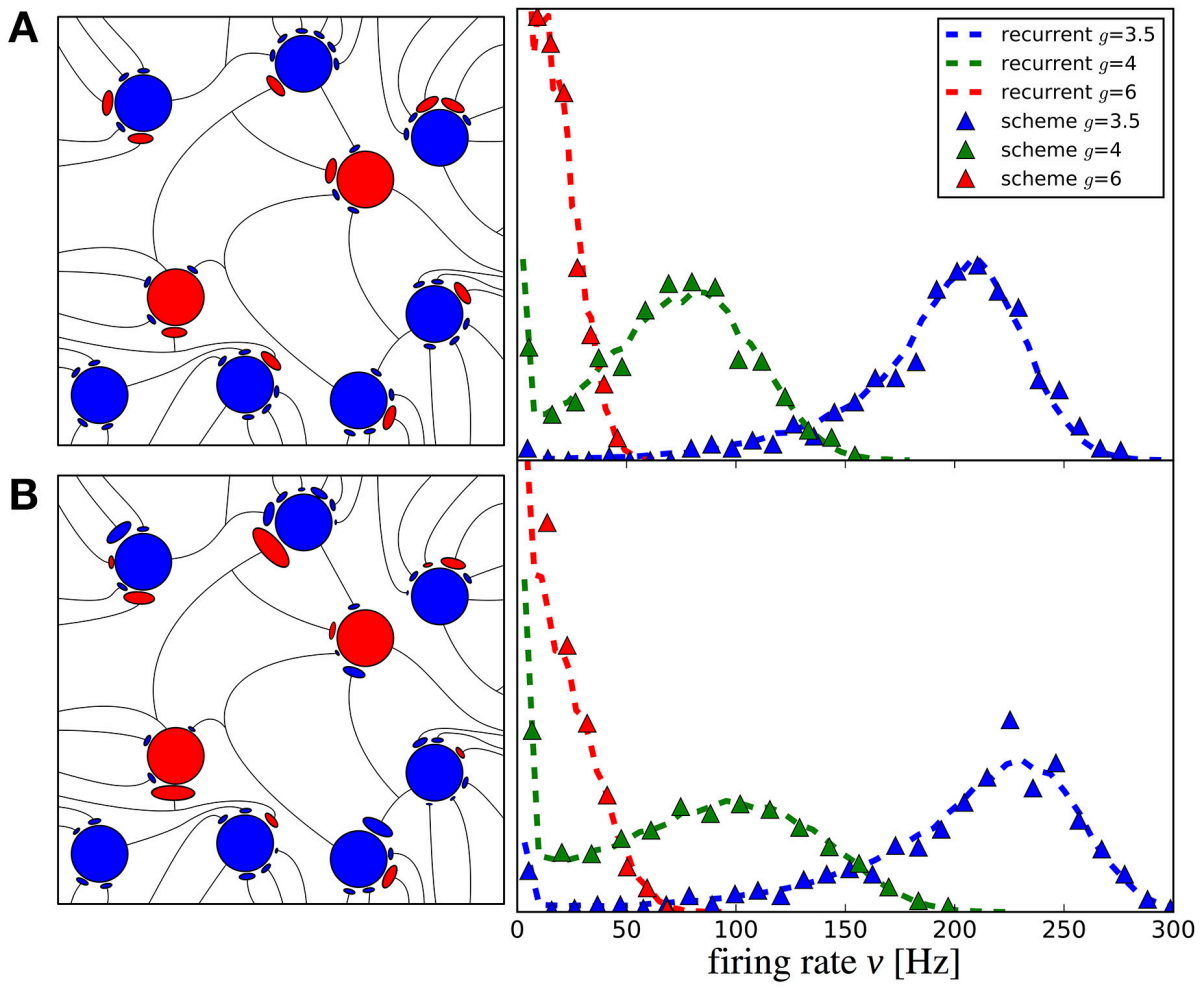
Iterative scheme reproduces firing rate distributions of Erdős-Rényi networks. Firing rate histograms of the iterative scheme with *M* = 50 neurons per generation (symbols) and the network simulation (dashed lines) for **(A)** binomially distributed numbers of presynaptic neurons [all excitatory (inhibitory) weights are the same as indicated on the left] and **(B)** additionally randomized synaptic weights (weights are drawn from distributions, sketched on the left). To build the histograms of the scheme, we used all firing rates from the 6th to the 20th generation (after transients have vanished). Parameters are as in Figure 10.

The first striking feature for a randomized connectivity is a broad distribution of firing rates. The stronger source for the rate variability seems to origin in the random number of connections (the case shown in Figure 9A); an exponential distribution of the synaptic strength makes the histograms only somewhat broader (case with additional weight variability, shown in Figure 9B). The histograms obtained from our iterative scheme with a modest number of *M* = 50 representative neurons (data are collected over several generations to improve the sampling) agree well with those from the network simulations. Note that analytical expressions for self-consistent distributions of the firing rate have been found previously using the diffusion approximation (Amit and Brunel, 1997; Roxin et al., 2011).

**Figure 10:**
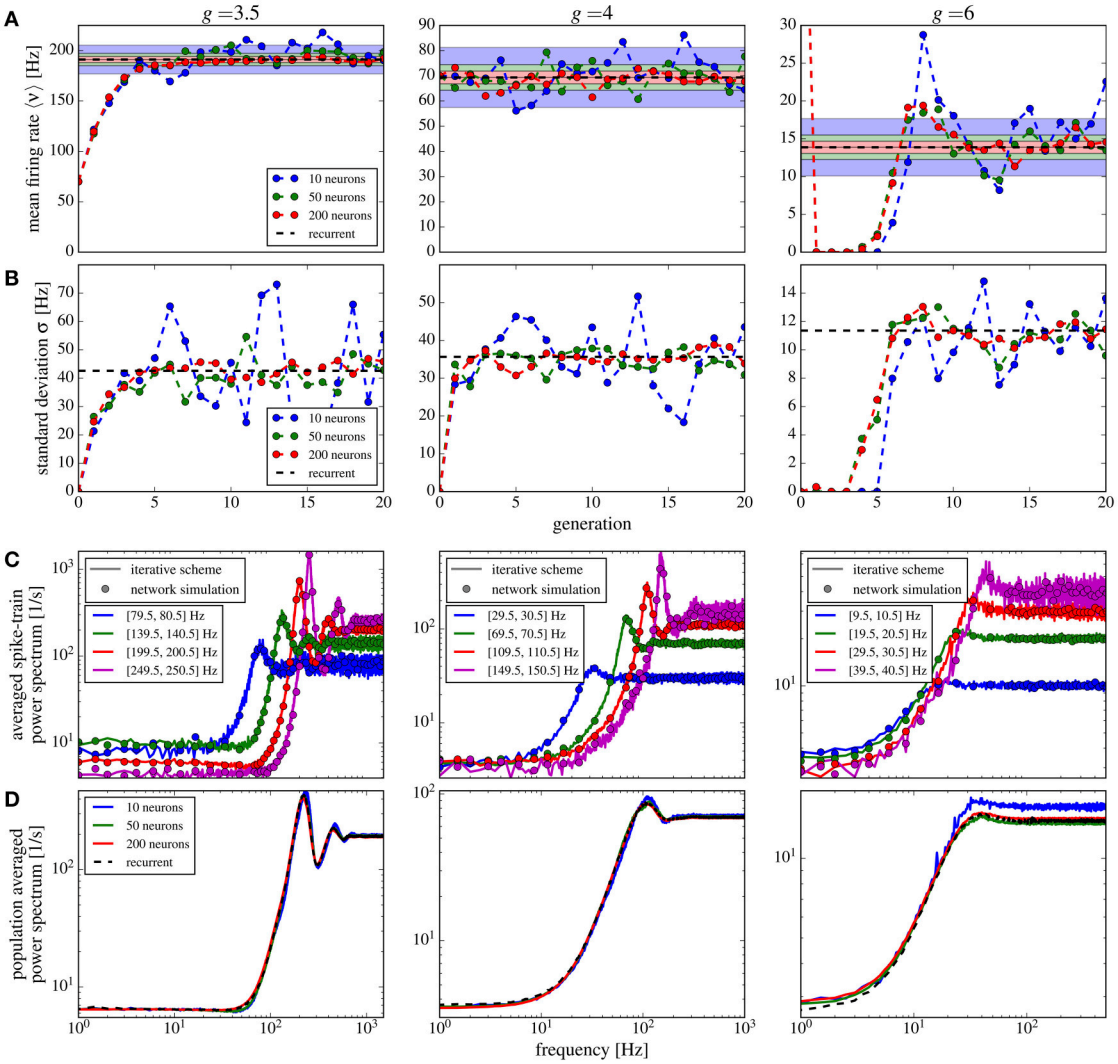
Iterative scheme captures the heterogeneous statistics due to Erdős-Rényi topology. Statistics from the iterative scheme and recurrent network for binomially distributed numbers of presynaptic neurons and different recurrent inhibition as indicated; remaining parameters: 〈*C*_*E*_〉 = 1000, 〈*C*_*I*_〉 = 250, *J* = 0.1 mV, τ_*m*_ = 20 ms, τ_*D*_ = 2 ms. For each neuron 100 trials of 10 s were used. From top to bottom: **(A)** mean firing rate for each generation of the scheme and the mean network rate (dashed line); standard error $\sigma_{s}=\sigma /\sqrt{M-1}$ (with σ being the standard deviation of the network firing rates) for different numbers of neurons *M* in the iterative scheme (colored areas). **(B)** Standard deviation of firing rates in the iterative scheme vs. generation compared to σ (dashed line). **(C)** Power spectra averaged over single neuron spectra with firing rates within the indicated intervals in the iterative scheme (lines, data from generations 6 to 20 for *g* = 3.5, 4 and 11–20 for *g* = 6) and from neurons in the network (circles). **(D)** Power spectra averaged over all neurons in the scheme (solid lines, data from generations 11 to 20) and network (dashed). For *g* = 6 we used the stabilization procedure from section 2.5.

In Figure 10 we show the mean Figure 10A and the standard deviation Figure 10B of the firing rate histograms of the iterative scheme as a function of the generation. Because the number *M* of neurons per generation (i.e., the number of samples) is finite we consider the standard error $\sigma_{s}=\sigma /\sqrt{M-1}$ represented by the colored areas in Figure 10A, where σ is the standard deviation of the firing rates measured from the network. The mean firing rate and standard deviation of the iterative scheme fluctuate around the network values within the expected standard error (colored areas), which indicates that the scheme works even for rather limited numbers of neurons *M* ≪ *N*. For the considered parameter sets, we observe fast convergence within five iterations for all used numbers of neurons except in the case of strong recurrent inhibition with *g* = 6 for which we observe convergence only after the 10th generation.

Power spectra are different for all neurons in the network and depend most strongly on the mean input that the respective cell receives. In the network we group neurons with similar firing rates (within a 1 Hz interval) and average their spectra within each group. We compare these spectra to those resulting from the self-consistent scheme having firing rates in the same interval (Figure 10C); we find a good agreement for a modest number of neurons *M* = 50 used in the scheme. Note that averaged spectra are very different from each other and from the average over all neurons in the network (Figure 10D). Even for a low number *M* = 10, the iterative scheme reproduces this average spectrum well.

**Figure 11:**
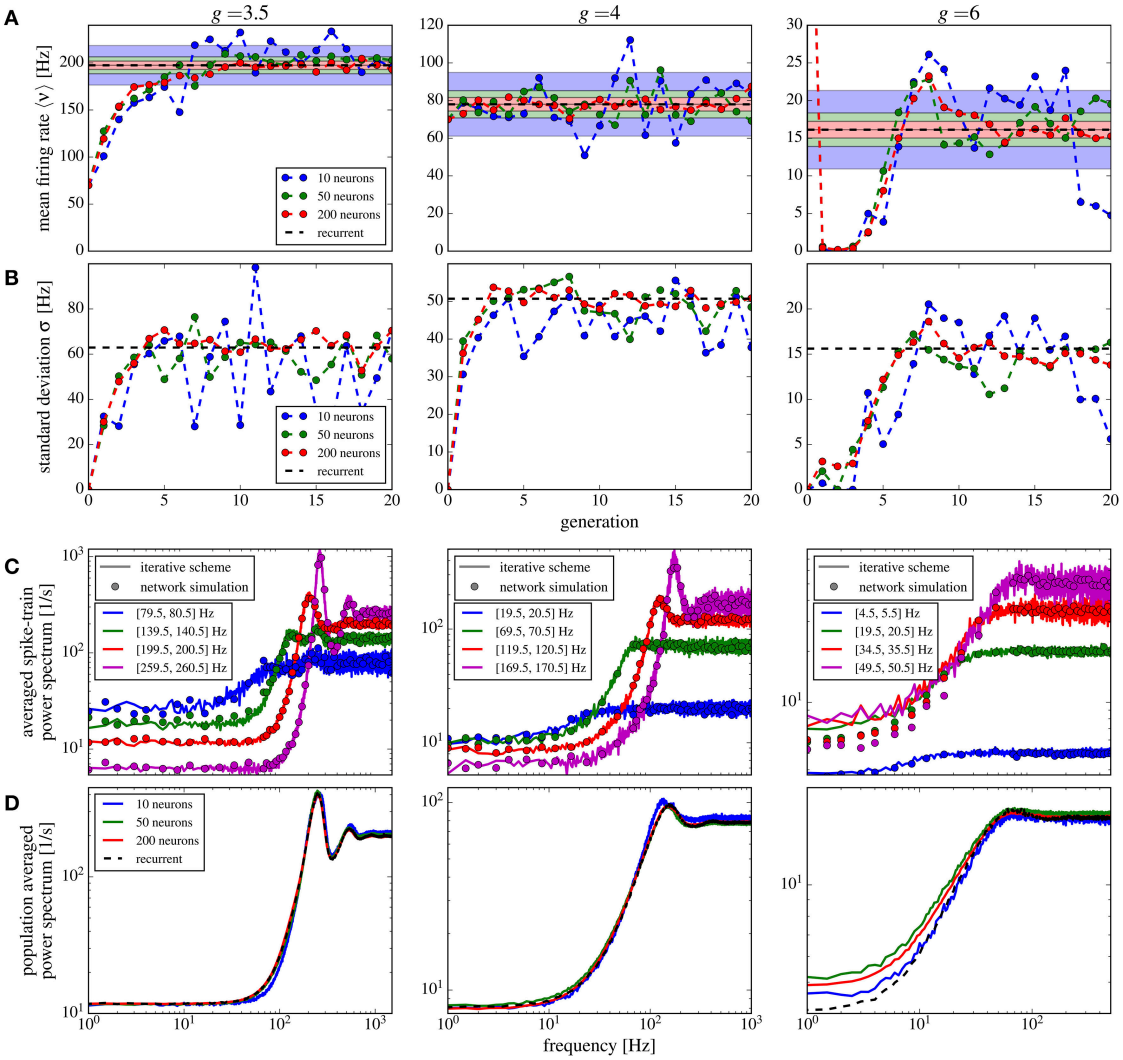
Iterative scheme captures the heterogeneous statistics due to Erdős-Rényi topology and randomly distributed synaptic weights. First- and second-order statistics from an iterative scheme for binomial distributed numbers of presynaptic neurons and additionally exponentially distributed synaptic weights. See caption of Figure 10 for details.

If we use in addition to the random number of connections also randomly distributed synaptic weights, the overall picture does not change qualitatively (Figure 11). Compared to the case of equal weights, the variance in the firing rates goes up by about 50% (Figure 11B) whereas the mean firing rate increases only slightly (Figure 11A). Power spectra look similar to the previous case and the agreement between scheme and network simulation is again good, except for the case of strong inhibition *g* = 6, for which we find a discrepancy between the averaged power spectra for the scheme and the network (Figure 11D) even for a large *M* (similarly for the single neuron spectra in Figure 11C). Due to the very low firing rates and some high synaptic weights, the Gaussian approximation becomes inaccurate. We calculated the relative error defined in Equation (19) for the averaged power spectra for *M* = 10 up to *M* = 100, using *f*_*cut*_ = 2〈ν〉. All observed relative errors were smaller than five percent.

The results of this section demonstrate how a relatively small number of neurons in the self-consistent scheme can be used to capture the first- and second-order statistics of neurons in a network with a variable number of inputs and synaptic weights.

### 3.5. Deviations due to non-gaussianity of current input

The iterative scheme assumes neuronal input that obeys a Gaussian distribution. This assumption is only accurate for a large number of pre-synaptic spikes with small amplitude. A low firing rate or an increase of (some) synaptic weights (as observed for stronger recurrent inhibition, i.e., larger values of *g*) shifts the statistics from approximately Gaussian to a distribution with pronounced non-Gaussian features (skewed and with fat tails). In fact, even the most basic statistics of the output, the firing rate, can systematically deviate from the Gaussian setting if synaptic amplitudes are sufficiently large (Richardson and Swarbrick, 2010). We can thus expect that at large values of *g* the iterative scheme (that assumes Gaussianity) may provide power spectra that deviate from those measured in the network; indeed, this is what we observed above. Here we want to illustrate the relation between the deviations of power spectra in the network and for the isolated neuron on the one side and non-Gaussian features of the network input statistics on the other side.

We simulate the heterogeneous network from section 3.4 with exponentially distributed weights and either *g* = 4 (as in Figure 11, middle column) or *g* = 6 (as in Figure 11, right column). We pick out 1,000 representative neurons randomly. For each of these neurons we measure the input current spectrum and the output spike-train spectrum. Input currents are integrated over a time bin of 2 ms (10% of the membrane time constant), in which the membrane voltage does not change too drastically but we can expect to collect many input spikes and thus Gaussian statistics. Clearly, for a much smaller time bin of the order of ${\left[\left(C_{E}+C_{I}\right)\bar{\nu }\right]}^{-1}\approx$ 0.01 ms (where $\bar{\nu }$ is the average firing rate of the presynaptic neurons), one single spike at most would typically fall into one time bin. Thus, on a very fine temporal scale, the input has *always* a highly non-Gaussian statistics. Nevertheless, the Gaussian approximation works well if the temporally integrated input noise has approximately Gaussian statistics for an effective time step, in which the voltage does not change much. As the deterministic drift causes already a change by several mV over a time of 2 ms, our choice of the effective time step is conservative in the sense that non-Gaussian features of the input in this time step will certainly become noticeable in the output statistics of the driven cell.

For each of the 1,000 selected neurons, we first measure the skewness and excess kurtosis of its input current to quantify non-Gaussianity. Then, we generate a Gaussian noise with the same power spectrum as the input current to this specific neuron and use it to drive an isolated LIF neuron. Finally, we calculate the relative error, Equation (19), between the output power spectrum of this isolated neuron and the corresponding neuron from the recurrent network. Note that for this comparison, we do not iterate the self-consistent scheme but rather use a Gaussian version of the (neuron-specific) network input noise for the isolated cell. In this way, we isolate non-Gaussianity of the input noise as one potential source of deviation between network spectra and spectra from the self-consistent scheme.

**Figure 12:**
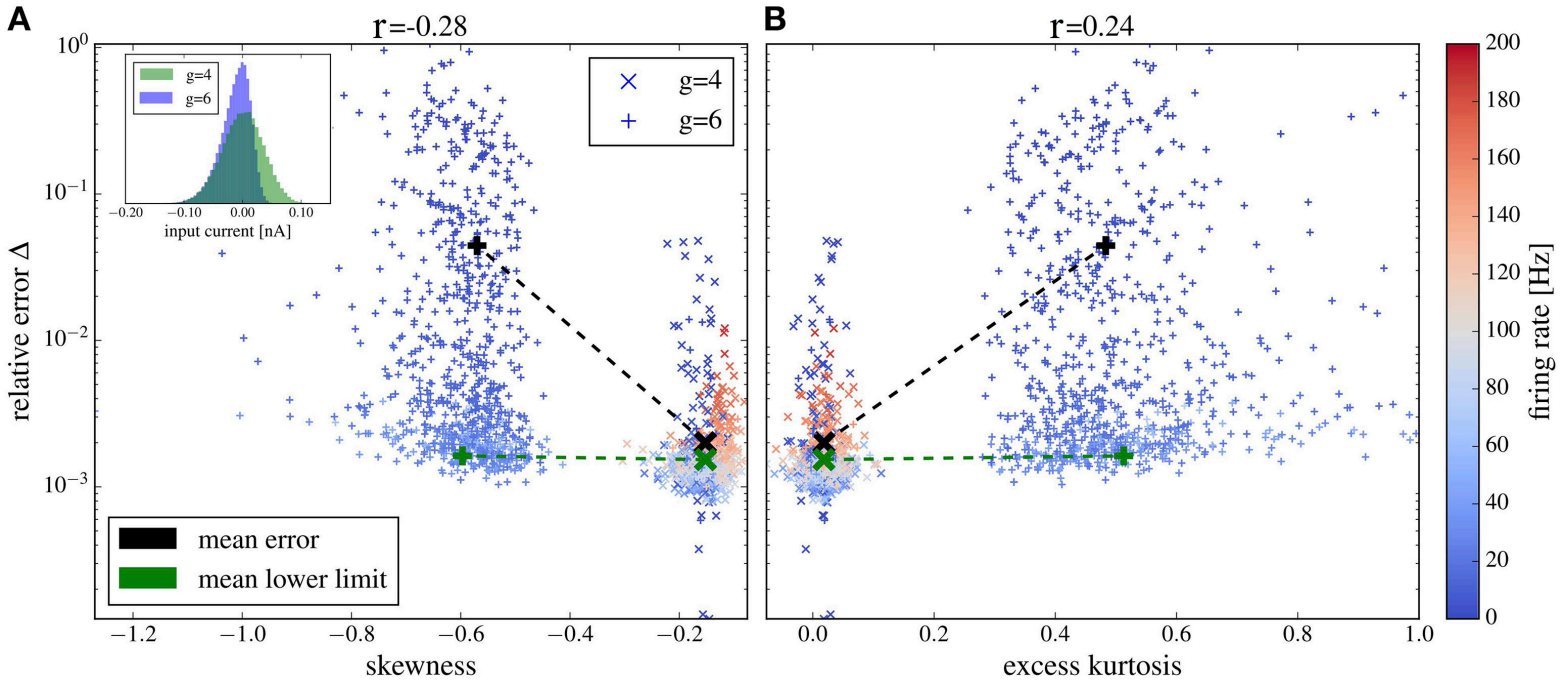
Deviation between spectra vs. statistical measures of non-Gaussianity of the input current. Relative error, Equation (19), of spike-train spectra from neurons in the network and from neurons driven by corresponding Gaussian noise plotted vs. skewness **(A)** and excess kurtosis **(B)** of the input current. Inset in **(A)** probability distributions for *g* = 4 and *g* = 6 as indicated. Color in **(A,B)** encodes the firing rate of each neuron (cf. scale on the right). We used the heterogeneous network from section 3.4 with exponentially distributed synaptic weights and *g* = 4 (crosses) and *g* = 6 (plusses) and picked 1,000 representative neurons randomly. For these neurons, we determined the skewness and excess kurtosis as well as the power spectrum of the input current. For the former two, we used the input current integrated over an effective time step of 2 ms (see main text for a discussion, how to choose this time step). We computed the relative error for each spike train-power spectrum from the network and its corresponding spike-train spectrum for the isolated neuron driven by the Gaussian version of the input noise (mean values shown by black symbols). The Pearson correlation coefficients are shown atop and indicate that deviations from Gaussian input statistics and deviations between network and single-neuron spectra co-occur. As a lower limit of the relative error, we computed Δ for two independent measurements of the power spectrum of a network neuron; this lower bound for the specific cell was then averaged over all neurons in the network and is shown by green crosses for both networks with *g* = 4 and *g* = 6 (there is no significant dependence of the mean lower bound on *g*).

Plots of the relative error vs. skewness and excess kurtosis are presented in Figures 12A,B, respectively. For *g* = 4 (balanced case, moderate inhibitory synaptic amplitudes), the relative error is generally small and skewness and excess kurtosis deviate only little from zero. In contrast, for stronger inhibitory synaptic weigths *g* = 6 (inhibition-dominated recurrent feedback), the input current has a pronounced negative skew and fat tails because of large inhibitory amplitudes, resulting in stronger deviations between the spectra of isolated neurons and the corresponding network neurons. Consequently, we find a correlation between non-Gaussianity and relative error (cf. the Pearson correlation coefficients given atop of the figure). To gauge the relative error, we need a lower limit. To this end, we consider the deviation between two measurements of the same power spectrum for a given neuron of the network. The average of this lower bound over the 1,000 selected neurons is shown in Figure 12 for comparison. It is close to the mean error for *g* = 4 but is significantly lower than the mean error for *g* = 6, and it barely depends on the skewness and kurtosis, again confirming the relation between non-Gaussianiaty of the input noise and the relative error between isolated neuron and network neuron.

**Figure 13:**
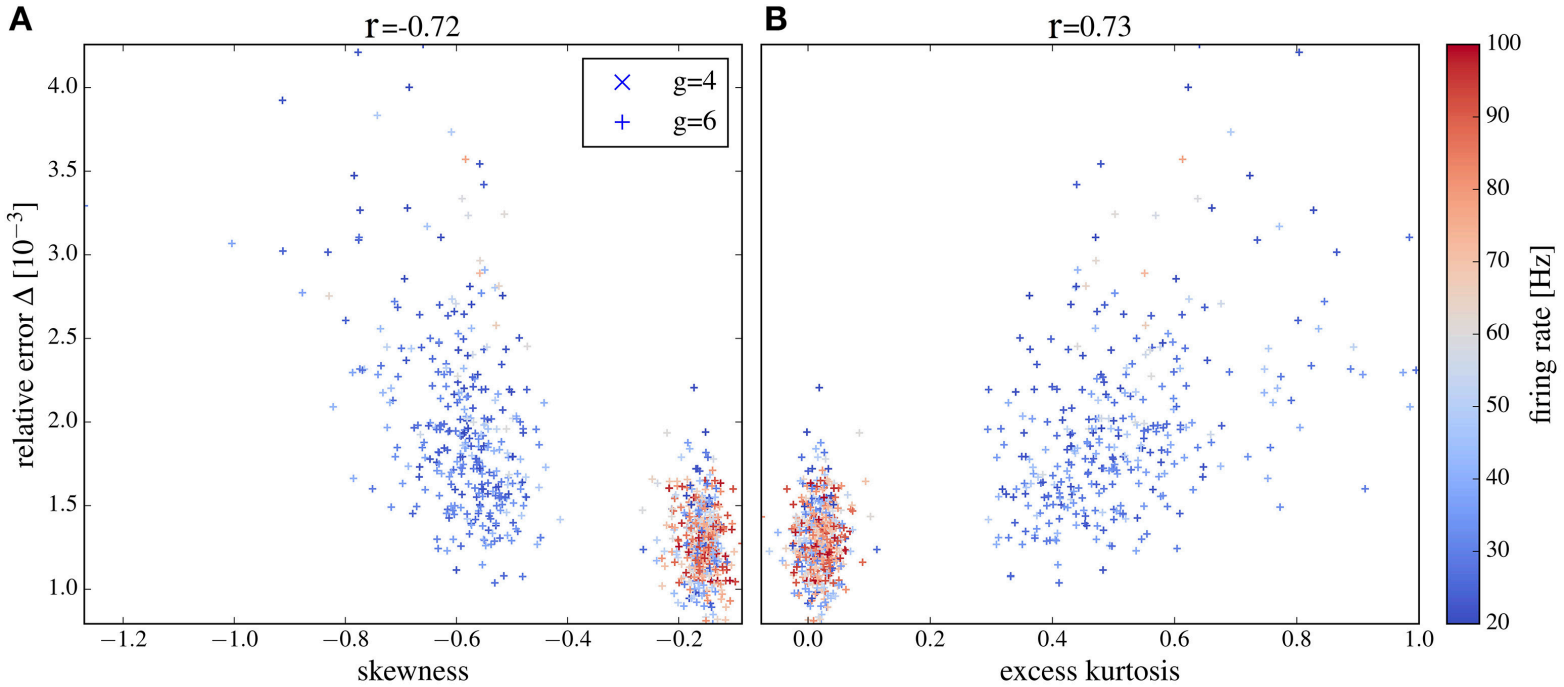
Deviation between spectra vs. statistical measures of non-Gaussianity of the input current for an intermediate range of output firing rates. Correlations between the relative error and skewness **(A)** and excess **(B)** are higher than in Figure 12 if only neurons with firing rates from 20 to 100 Hz are selected. Other parameters as in Figure 12.

For both values of *g* we obtain a high relative error for exceptionally large firing rates (>100Hz) (because the time step of 2 ms becomes too large in comparison to the mean ISI) or exceptionally low firing rate (because spectra are very noisy in this case). If we consider only a range of moderate firing rates (Figure 13) then the correlation between the deviation from Gaussianity and the discrepancy between network and isolated becomes even clearer (cf. Pearson correlations coefficients stated atop of the figure).

## 4. Conclusions

In this work we extended the self-consistent scheme described by Dummer et al. (2014) to situations with strong inhibition, synaptic filtering, networks with subpopulations of distinct neuron types, and networks with random (instead of constant) number and strength of synaptic connections. In all cases we employed the Gaussian approximation and in the heterogeneous systems considered we used a small number of neurons each representing a certain subclass of similar neurons. Despite these approximations, our comparison of the determined spike-train power spectra with those found by numerical simulations of large and sparse recurrent networks revealed a good quantitative agreement.

Admittedly, with an increasing number of subtypes of neurons, we loose some of the numerical advantages of the scheme compared to a network simulation because in order to get reliable estimates of the power spectrum, we have to simulate the few neurons in each generation many times. If the convergence of the scheme is slow then, adding up all neurons in all generations and all trials, we may have to simulate in the end as many neurons as in the network (however, the typical bottleneck of many simulations, to keep track of all synaptic connections, is still absent in the scheme). It is thus questionable, whether much more complicated situations than discussed here can be studied in depth by our scheme.

Another short-coming of the approach concerns cases in which neural cross-correlations (a very vivid topic of current research, see Doiron et al., 2016) cannot be neglected anymore or in which weak cross-correlations still have a significant impact on the population activity (Schneidman et al., 2006). There are different causes for cross-correlations including common (shared) input, spatially homogeneous external stimuli, and a slight overall synchronization in the network (some of which are reviewed by Helias et al., 2014; Doiron et al., 2016). Not all of these factors can be taken into account by extending the scheme to pairs of neurons that are stimulated by correlated Gaussian noise processes^2^. We may still learn something from finding situations in which neural cross-correlations can quantitatively be described by extensions of the scheme to pairs of neurons in each generation.

Nevertheless, the results and the approach put forward in our paper are useful in several respects. If the single-neuron statistics is of interest (because this is what is recorded or this is what shows particularly interesting features), our method provides a computationally cheap solution to calculate the spike-train power spectrum and to study its dependence on cellular and network parameters without the need to simulate a network. The scheme is particularly suited for the idealized case of a perfectly asynchronous network that is difficult to study numerically because an almost completely asynchronous state can be reached only in a very sparse, hence, very large network. This case is interesting because it often permits analytical calculations via a density equation for the membrane voltage (Knight, 1972; Abbott and van Vreeswijk, 1993; Amit and Brunel, 1997; Brunel, 2000; Mattia and Giudice, 2002) and thus our scheme might be useful for comparison to simpler theories.

As already mentioned in the introduction, we can regard our results as a confirmation that the approximation of the synaptic input by a correlated Gaussian noise is a reasonable one over a physiological range of parameters for a sparse recurrent network in the asynchronous state. Using Markovian embedding, an arbitrary colored Gaussian noise can be described by a (possibly very high-dimensional) Ornstein-Uhlenbeck process, an idea that has been worked out in the neural context by Schwalger et al. (2015); for examples from the physics literature, see, for instance, Schimansky-Geier and Zülicke (1990); Hänggi and Jung (1995); Siegle et al. (2010). Hence, a stochastic mean-field theory in terms of the corresponding multidimensional Fokker-Planck equation seems to be in reach, generalizing the successful framework of the diffusion approximation, which was based on the Poissonian (white-noise) approximation and thus led to a one-dimensional Fokker-Planck equation. A theory using the colored-noise Fokker-Planck equation would faithfully reproduce the second-order temporal correlations of the spiking neurons and, possibly, provide novel insights into the bifurcation between asynchronous and synchronous states. This may be particularly relevant for larger synaptic amplitudes (Ostojic, 2014; Wieland et al., 2015), for which the color of the noise becomes more and more important.

## Author contributions

RP, SV, DB, ACR, and BL: Conceived the work; RP and SV: Developed the codes and performed the computations; RP, SV, DB, and BL: Analyzed the results; RP, SV, DB, ACR, and BL: Wrote the manuscript.

### Conflict of interest statement

The authors declare that the research was conducted in the absence of any commercial or financial relationships that could be construed as a potential conflict of interest.

## Footnotes

^1^This is evident for a finite synaptic filter, that leads to a summation of many independent continuous functions. For delta synapses, we add up spike trains and so it becomes questionable, how they can approach a Gaussian statistics. In this latter case we should consider the effect of the summed shot noise on a dynamical system such as the integrate-and-fire neuron: the sum over a small time interval (smaller than the membrane time constant but large enough to collect many independent spikes from the presynaptic neurons) yields a spike count which can be well approximated by a Gaussian variable; this is similar to the common diffusion approximation (Tuckwell, 1988), which, however, additionally involves the assumption of a Poissonian input spike train. In this sense, the Gaussian approximation can be applied to sums of spike trains (see also Figure 12 and the surrounding discussion, for a test of the Gaussianity of the input current).

^2^For the simpler but still formidable problem of how neuron pairs respond to cross-correlated Gaussian white noise sources, see, for instance, Doiron et al. (2004); de la Rocha et al. (2007); Shea-Brown et al. (2008); Ostojic et al. (2009); Vilela and Lindner (2009b); Deniz and Rotter (2017).
